# Convolutional Neural Network Models for Visual Classification of Pressure Ulcer Stages: Cross-Sectional Study

**DOI:** 10.2196/62774

**Published:** 2025-03-25

**Authors:** Changbin Lei, Yan Jiang, Ke Xu, Shanshan Liu, Hua Cao, Cong Wang

**Affiliations:** 1 Trauma Center, West China Hospital, West China School of Nursing, Sichuan University, Chengdu, China; 2 Nursing Department, Evidence-Based Nursing Center, West China Hospital, Sichuan University, Chengdu, China; 3 Evidence-Based Nursing Center, West China Hospital, Sichuan University, Chengdu, China; 4 Neurosurgery Department, West China Hospital, Sichuan University, Chengdu, China

**Keywords:** pressure ulcer, deep learning, artificial intelligence, neural network, CNN, machine learning, image, imaging, classification, ulcer, sore, pressure, wound, skin

## Abstract

**Background:**

Pressure injuries (PIs) pose a negative health impact and a substantial economic burden on patients and society. Accurate staging is crucial for treating PIs. Owing to the diversity in the clinical manifestations of PIs and the lack of objective biochemical and pathological examinations, accurate staging of PIs is a major challenge. The deep learning algorithm, which uses convolutional neural networks (CNNs), has demonstrated exceptional classification performance in the intricate domain of skin diseases and wounds and has the potential to improve the staging accuracy of PIs.

**Objective:**

We explored the potential of applying AlexNet, VGGNet16, ResNet18, and DenseNet121 to PI staging, aiming to provide an effective tool to assist in staging.

**Methods:**

PI images from patients—including those with stage I, stage II, stage III, stage IV, unstageable, and suspected deep tissue injury (SDTI)—were collected at a tertiary hospital in China. Additionally, we augmented the PI data by cropping and flipping the PI images 9 times. The collected images were then divided into training, validation, and test sets at a ratio of 8:1:1. We subsequently trained them via AlexNet, VGGNet16, ResNet18, and DenseNet121 to develop staging models.

**Results:**

We collected 853 raw PI images with the following distributions across stages: stage I (n=148), stage II (n=121), stage III (n=216), stage IV (n=110), unstageable (n=128), and SDTI (n=130). A total of 7677 images were obtained after data augmentation. Among all the CNN models, DenseNet121 demonstrated the highest overall accuracy of 93.71%. The classification performances of AlexNet, VGGNet16, and ResNet18 exhibited overall accuracies of 87.74%, 82.42%, and 92.42%, respectively.

**Conclusions:**

The CNN-based models demonstrated strong classification ability for PI images, which might promote highly efficient, intelligent PI staging methods. In the future, the models can be compared with nurses with different levels of experience to further verify the clinical application effect.

## Introduction

Pressure injuries (PIs), also known as pressure ulcers, are prevalent in health care settings and result from sustained pressure or shear forces on bony prominences and soft tissues [1]. A meta-analysis encompassing 2,579,049 hospitalized adult patients revealed a 12.8% prevalence of PIs, and the estimated annual total cost of managing PIs increased approximately several times even in high-income countries [2,3]. Accurate staging of PIs is essential for determining appropriate treatment protocols and predicting clinical outcomes. Traditional staging relies on subjective evaluation by health care providers, often wound nurses, who use guidelines such as the *Prevention and Treatment of Pressure Ulcers/Injuries: Clinical Practice Guideline, International Guideline 2019*, and divide the PI stage into 6 stages—namely, stages I, II, III, and IV, unstageable, and suspected deep tissue injury (SDTI)—based on visual assessment, such as the color and texture characteristics of the wound bed, wound edges, and skin around the wound [4].

However, visual assessment conducted by medical staff is subject to considerable interrater and intrarater variability [5]. Moreover, the staging results are significantly influenced by the level of wound knowledge and the clinical experience of the evaluators. It has been reported that only 23%-58% of medical staff correctly classify PIs [6]. Therefore, accurate staging of PIs is still a great challenge, and a universal, reliable, and more objective staging system is urgently needed.

Convolutional neural networks (CNNs) offer an opportunity to increase the objectivity and accuracy of PI staging. CNNs, a subset of deep learning (DL) models, have shown remarkable potential in medical image analysis [7]. CNNs automatically extract and learn hierarchical features from grid-like data, such as images, and have achieved performance levels comparable to or surpassing those of human experts in various medical imaging domains [8]. In dermatological and wound imaging, CNNs have demonstrated promising results, matching or even exceeding the diagnostic accuracy of dermatologists in classifying skin cancer and other skin lesions [9]. On the basis of the good classification performance of CNNs in medical images and the increased clinical need for automated staging of PIs, in recent years, the use of CNNs to learn PI images has gradually developed, but there are also areas for improvement. For example, the use of a singular model had an accuracy rate below 90%, alongside images sourced from outdated databases and of poor quality, and the labeling of PI images often relies on the subjective judgment of clinical experts, so the quality and consistency of the images were uneven in these studies, which limit the evidence supporting the model’s widespread applicability [10-12]. Therefore, further exploration is needed in this field to prove the effectiveness of CNNs.

In this study, we trained AlexNet, VGGNet16, ResNet18, and DenseNet121 to classify PI images with the aim of contributing to artificial intelligence–driven wound care knowledge and informing the development of advanced clinical tools.

## Methods

### Setting and Samples

Our study was conducted at a tertiary hospital in Chengdu, China, from March 1, 2022, to September 30, 2024.

The inclusion criteria for patients were as follows: (1) PIs were diagnosed by 2 wound therapists simultaneously on the basis of the *Prevention and Treatment of Pressure Ulcers/Injuries: Clinical Practice Guideline, International Guideline 2019* [4] and (2) the patients provided informed consent.

The exclusion criteria for patients were as follows: (1) PIs included other skin diseases, such as incontinence dermatitis; (2) PIs were covered by dressings or tattoos; (3) exudation was excessive, resulting in obvious reflection; (4) mucosal PIs; (5) the patient’s vital signs remained too unstable to exchange positions; and (6) the patients were in isolation wards.

The inclusion criteria for images were as follows: (1) images were taken by the investigator, and (2) images fully presented the wound triangle, namely, the wound bed, wound edge, and surrounding wound tissue.

The exclusion criteria for images were as follows: (1) images containing nonwounded and skin tissues such as clothing and sheets and (2) images that were blurred and overexposed.

### Dataset

We recruited wound therapists who were awarded an international ostomy wound certificate to act as PI staging evaluators. Five wound therapists were included in this study. The basic information is provided in Table 1. To test the consistency of the 5 wound therapists’ assessments, we sent them 30 PI images from the NPIAP (National Pressure Injury Advisory Panel) website in the form of a questionnaire [13]. The Fleiss kappa coefficient was 0.856, *P*<.001, indicating strong staging consistency among them [14]. The staging results are shown in Tables2  3.

**Table 1.** Basic information of the 5 wound therapists.

Order	Gender	Age (years)	Working years	Department
A	Female	42	20	Respiratory ICU^a^
B	Female	36	15	Neurology
C	Female	40	18	General ICU
D	Female	43	25	Neurology
E	Female	36	15	ICU

a ICU: intensive care unit.

**Table 2.** Pressure injury staging results of the 5 wound therapists.

Order	Staging results of the 5 wound therapists				
	A	B	C	D	E
1	Stage III	Stage III	Stage III	Stage III	Stage IV
2	Stage I	Stage I	Stage I	Stage I	Stage I
3	Stage II	Stage III	Stage II	Stage II	Stage II
4	Unstageable	Unstageable	Stage IV	Unstageable	Unstageable
5	Unstageable	Unstageable	Unstageable	Unstageable	Unstageable
6	Stage IV	Stage IV	Stage IV	Stage IV	Stage IV
7	SDTI^a^	SDTI	SDTI	SDTI	SDTI
8	Stage I	Stage I	Stage I	Unstageable	Stage I
9	Stage IV	Stage IV	Stage IV	Stage IV	Stage IV
10	Unstageable	Stage IV	Stage IV	Stage IV	Stage IV
11	Stage III	Stage III	Stage III	Stage III	Stage III
12	Stage III	Stage III	Stage III	Stage III	Stage III
13	Stage II	Stage III	Stage II	Stage II	Stage II
14	SDTI	SDTI	SDTI	SDTI	SDTI
15	Stage IV	Stage IV	Stage IV	Stage IV	Stage IV
16	SDTI	SDTI	SDTI	SDTI	SDTI
17	Stage I	Stage I	Stage I	Stage I	Stage I
18	Stage II	Stage III	Stage II	Stage II	Stage II
19	Stage III	Stage III	Stage III	Stage III	Stage III
20	Stage IV	Stage SDTI	Stage IV	Stage IV	Stage IV
21	Stage I	Stage I	Stage I	Stage I	Stage I
22	Unstageable	Unstageable	Unstageable	Unstageable	Unstageable
23	SDTI	SDTI	SDTI	SDTI	SDTI
24	Stage II	Stage II	Stage II	Stage II	Stage II
25	Stage I	Stage I	Stage I	Stage I	Stage I
26	Stage II	Stage II	Stage II	Stage II	Stage II
27	Unstageable	Unstageable	Unstageable	Unstageable	Unstageable
28	Stage IV	Stage IV	Stage IV	Stage IV	Stage IV
29	Stage III	Stage III	Stage III	Stage III	Stage III
30	SDTI	SDTI	SDTI	I	SDTI

a SDTI: suspected deep tissue injury.

**Table 3.** Coefficient of internal consistency among the 5 wound therapists. The sample data contain 30 valid participants and 5 raters.

		Asymptotic			
	Kappa	SE	*Z*	*P* value	Asymptotic 95% CI
Overall	0.856	0.026	32.948	<.001	0.805-0.906

### The PI Staging Assessment Process and Shooting Process

Before taking the shots, the researcher and the 2 wound therapists communicated with the patients and their family members to obtain consent. After that, the 2 therapists simultaneously evaluated the staging of the pressure ulcers via the *Prevention and Treatment of Pressure Ulcers/Injuries: Clinical Practice Guideline, International Guideline 2019*. If there were any objections to the results, we informed the third wound therapist on duty, and they would negotiate the staging.

The following types of shooting equipment were used: (1) Fuji XT-4 was used for shooting, with 21.6 million effective pixels, and Fuji XF 60 mm F2.4 R Macro and (2) a gray card. The specifications of the shooting mode are as follows: (1) antishake plus automatic mode; (2) parameter setting: shutter time: 60/1 s; (3) sensitivity: 3600-6000; (4) aperture: F6-8; (5) focal length: 60 mm; and (6) white balance: automatic white balance.

**Figure 1.**
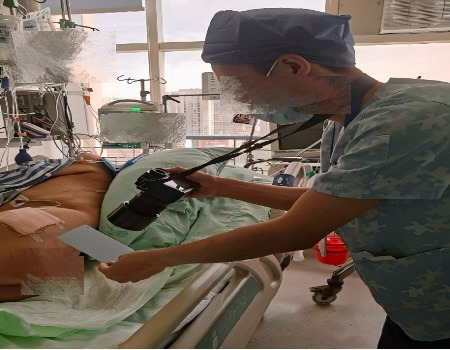
Shooting process.

When photographs were taken, the camera was positioned parallel to the wound, with a focus on both the wound itself and the surrounding skin (Figure 1). Additionally, a gray card was used to minimize any interference caused by natural light. Following each shot, the images were carefully examined. If any image was deemed unclear, the researcher retracted the image. All of the original images were saved in JPG format and given a specific name consisting of a stage followed by a corresponding number. Finally, the images were transferred onto a computer for further analysis and storage.

### Image Augmentation

**Figure 2.**
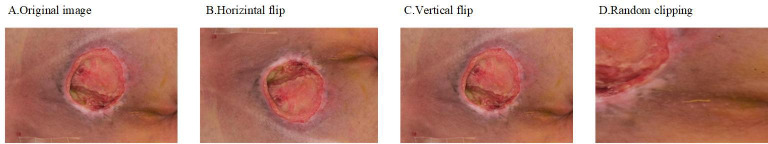
Image augmentation: (A) original image, (B) horizontal flip, (C) vertical flip, and (D) random clipping.

Horizontal flipping, vertical flipping, and random clipping were used for data augmentation to expand the training dataset and improve the generalization ability of the models (Figure 2) [15]. Taking random cropping as an example, the size of the original image was reset to 512×512×3, and after random cropping, the size was 256×256×3. The size of the augmented image was fixed again to 224×224×3 before being input into the networks so that the network model could recognize them.

### Image Normalization

**Figure 3.**
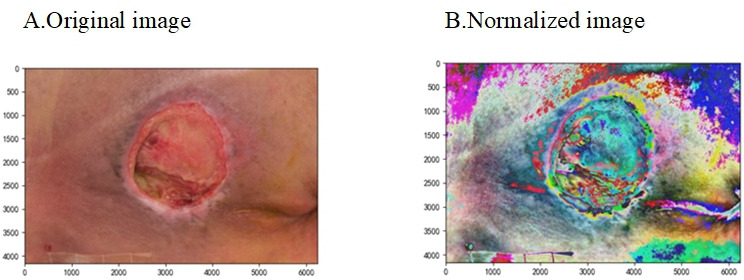
Image normalization: (A) original image and (B) normalized image.

The PI images are RGB color patterns (Figure 3), and the number of pixels is [0, 256] [16]. To reduce the adverse effects caused by singular sample data and speed up model training, we limit the number of pixels to [−1, 1] in this study via 2 image normalization calculation formulas, where $\bar{x}$ is the mean of the number of pixels, *N* represents the number of pixels, and *x* represents the pixel value of each pixel [17]. The calculation formula is as follows:

(1)$s={\left(\frac{1}{N-1}\sum_{i=1}^{N}{\left(x_{i}-\bar{x}\right)}^{2}\right)}^{\frac{1}{2}}$

The Adam optimizer was used, and the loss function was the cross-entropy loss function (cross-entropy loss function), which is commonly used in multiple classification tasks. The data batch (batch size) was set to 8, and the initial learning rate was set to 0.0001. Every network was trained for 30 epochs.

This study was based on the 64-bit Windows 11 computer system and the Ubuntu 16.04 operating system for image training in the PyTorch 2.0.1 framework using the Python 3.9.19 language with CUDA 11.8 and NVIDIA GeForce RTX 4060 Laptop (8G).

**Figure 4.**
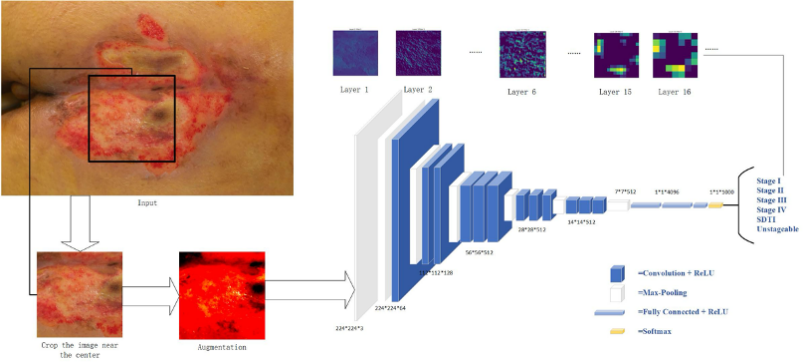
The network workflow diagrams. SDTI: suspected deep tissue injury.

The process of model training was divided into training, validation, and testing. The process is shown in Figure 4. First, the PI image was cut into the main wound area, and the cut image was treated as a uniform pixel, both of which were cut to 224 pixels. In the training set, the CNN training model is input; the performance of the model on the validation set is evaluated; and the state and convergence of the model are tested to adjust the hyperparameters. In the test set, the model outputs corresponding prediction results through a series of convolutions, nonlinear activations, pooling, etc, to evaluate the generalization ability of the model.

In our study, we used AlexNet, VGGNet 16, ResNet 18, and DenseNet121 to train the images. AlexNet, a pioneering CNN in image classification, consists of 8 layers: 5 convolutional layers with varying filter numbers and 3 fully connected layers. It employs ReLU activations, max pooling, and dropout for regularization. The introduction of ReLU and dropout layers in AlexNet reduced training times and prevented overfitting, whereas its deep architecture allowed for the learning of complex features, enhancing classification accuracy [18].

VGGNet 16 is a 16-layer deep network consisting of 13 convolutional layers and 3 fully connected layers, all of which use 3×3 filters. It features a consistent architecture with convolutional layers followed by max pooling layers, which simplifies optimization. This network is effective for capturing grid-like patterns in images and allows for the learning of more abstract and complex features due to its depth [19].

ResNet 18 is a shallow network with 18 layers organized in a residual learning framework. It consists of 4 blocks of residual units, each with convolutional layers, batch normalization, ReLU activation, and max pooling layers. The key innovation is the introduction of residual connections, which allow the network to learn residual mappings, making it easier to train deeper networks by effectively flowing gradients through the network [20].

DenseNet121 connects each layer to every other layer in a feedforward manner, using features from all preceding layers as inputs and its own feature map for all subsequent layers. This dense connectivity reduces the number of parameters, making DenseNet121 more parameter-efficient than other CNNs of similar depth. It helps mitigate the vanishing gradient problem and enhances feature propagation, leading to improved performance and efficiency [21].

In summary, the 4 CNN models were selected for their historical performance in image classification tasks, the uniqueness of their architectural design, and their significant contributions to the field of DL (Figure 4).

### Evaluation of Performance

We evaluated the performance from a single image result. The diagnostic performance was measured by accuracy (ACC), precision (Pre), recall (Rec), and the *F*_1_-score. To calculate the above metrics, we defined an abnormal result as positive and a normal result as negative.

(1) $\text{ACC}=\frac{\text{TP}+\text{TN}}{\text{TP}+\text{FP}+\text{FN}+\text{TN}}$

(2) $\text{Pre}=\frac{\text{TP}}{\text{TP}+\text{FP}}$

(3) $\text{Rec}=\frac{\text{TP}}{\text{TP}+\text{FN}}$

(4) $F1\text{-score}=2\cdot \frac{\text{Precision}\cdot \text{Recall}}{\text{Precision}+\text{Recall}}$

### Ethical Considerations

This study was approved by the Biomedical Ethics Committee of the West China Hospital of Sichuan University (#1053). In this study, the informed consent process was strictly carried out. Participation in the study was completely voluntary, and patients could refuse to participate or withdraw at any time during any phase of the study without discrimination or retaliation and without affecting their medical treatment and benefits. If participants decided to withdraw from the study, they would contact us. Patient privacy was strictly protected, and all data obtained were ony used for this study. Patients did not have to pay any fees to participate in the study.

## Results

**Figure 5.**
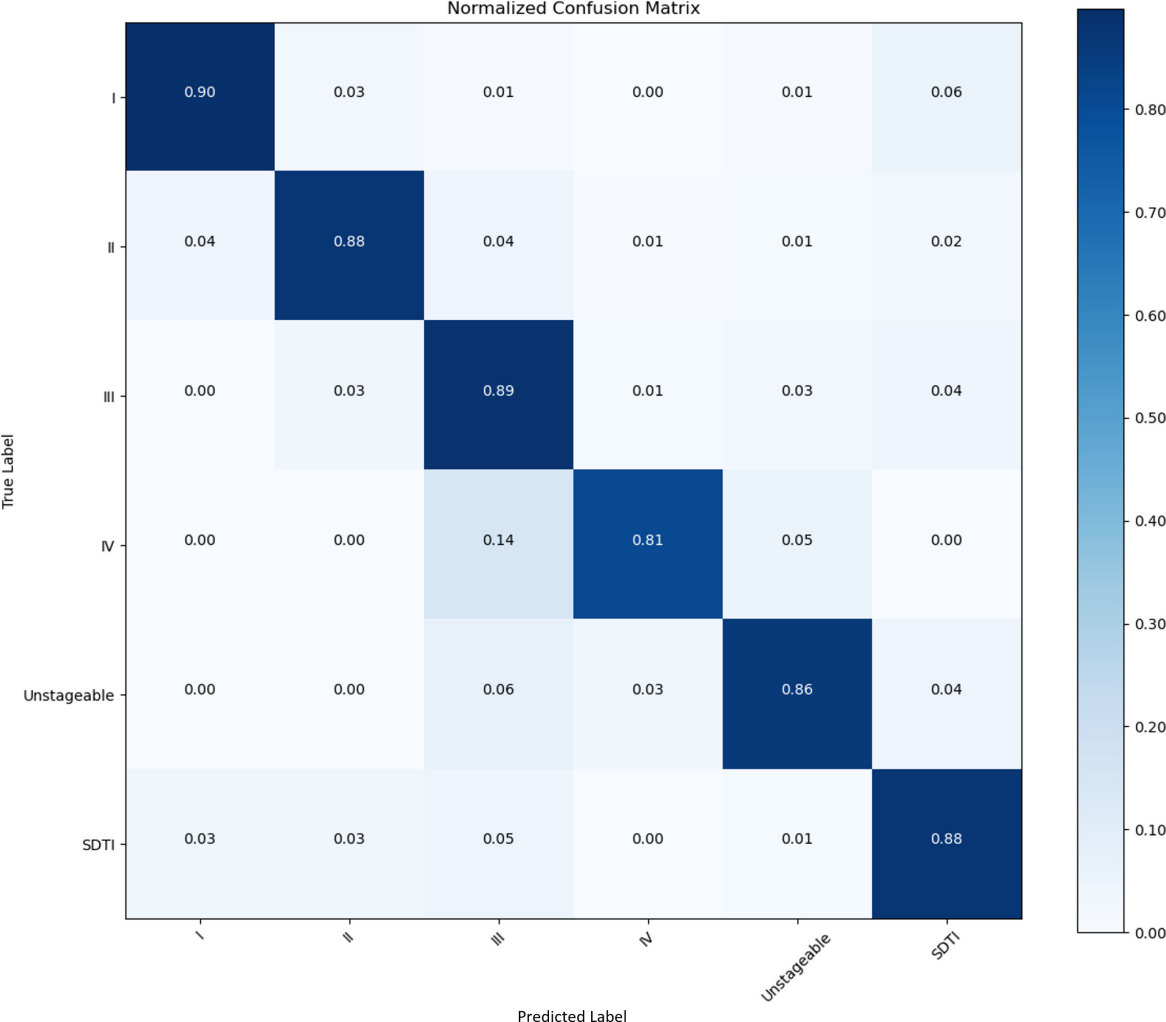
Performance of the CNNs in the identification and classification of PI images. Confusion matrix showing the accuracy and precision of 87.74% and 97.48%, respectively. CNN: convolutional neural network; PI: pressure injury; SDTI: suspected deep tissue injury.

**Figure 6.**
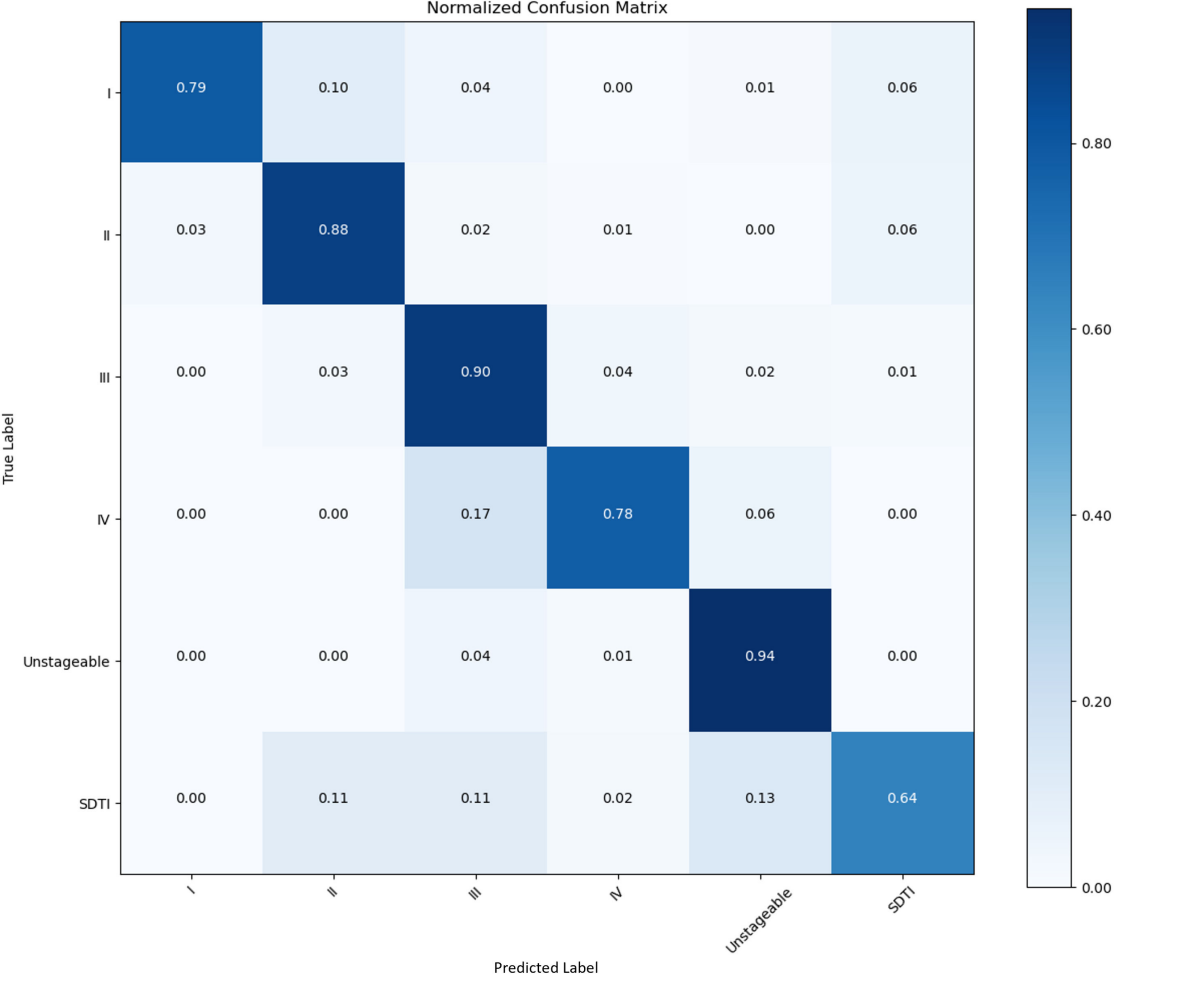
Performance of the CNNs in the identification and classification of PI images. Confusion matrix showing the accuracy and precision of 82.42% and 92.4%, respectively. CNN: convolutional neural network; PI: pressure injury; SDTI: suspected deep tissue injury.

**Figure 7.**
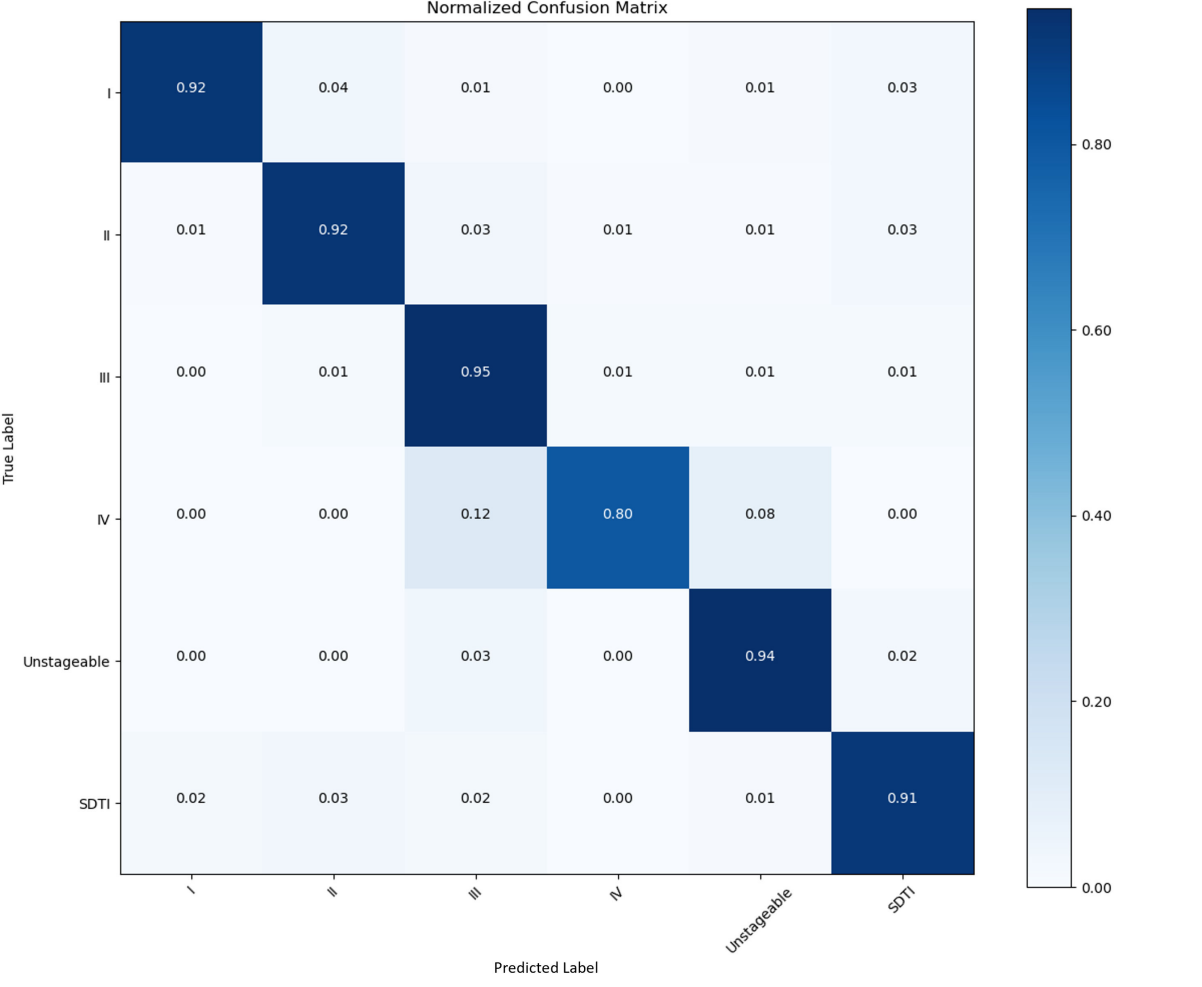
Performance of the CNNs in the identification and classification of PI images. Confusion matrix showing the accuracy and precision of 92.42% and 98.43%, respectively. CNN: convolutional neural network; PI: pressure injury; SDTI: suspected deep tissue injury.

**Figure 8.**
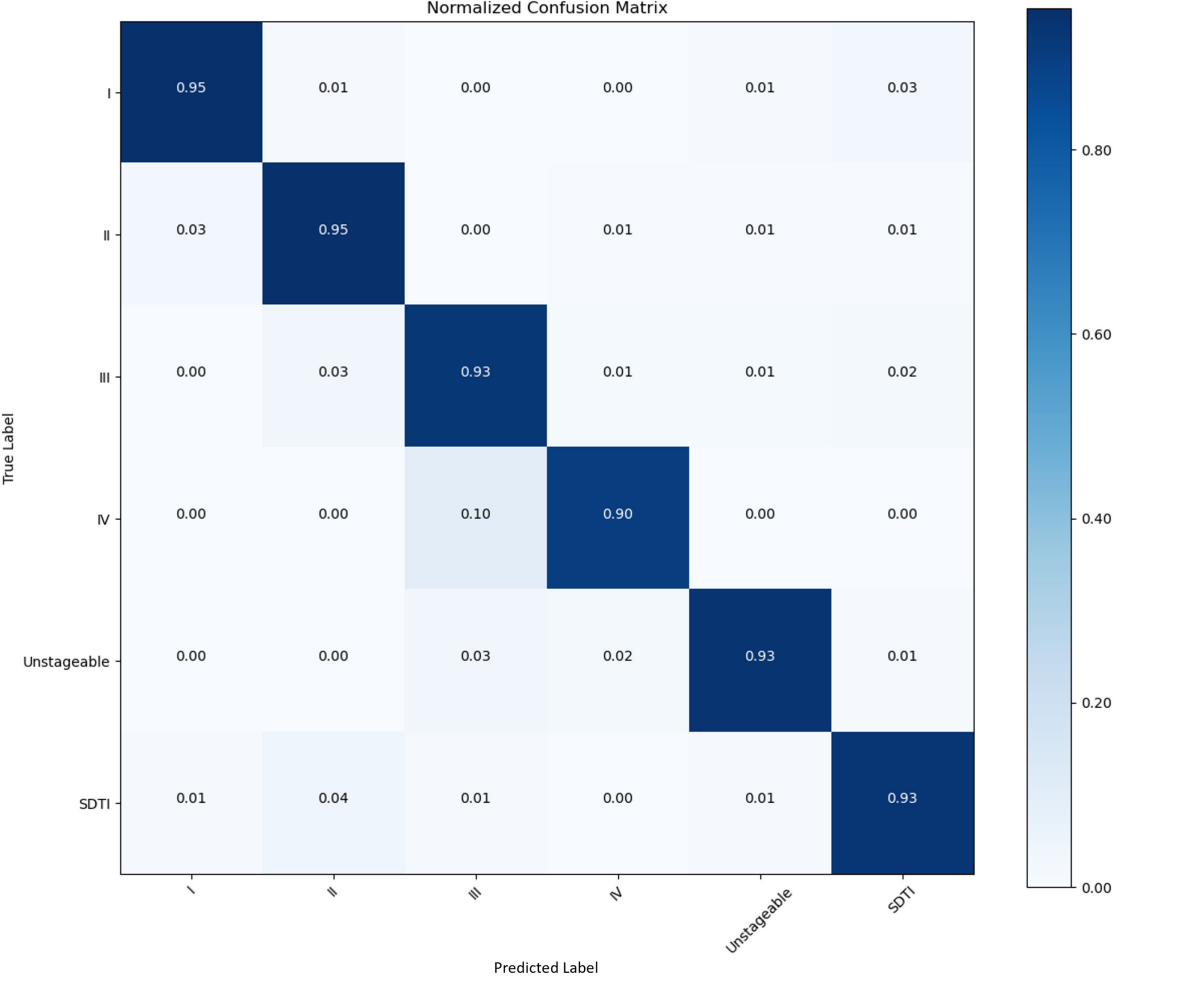
Performance of the CNNs in the identification and classification of PI images. Confusion matrix showing the accuracy and precision of 93.71% and 98.72%, respectively. CNN: convolutional neural network; PI: pressure injury; SDTI: suspected deep tissue injury.

We collected 853 raw PI images in this study. We set the images into a training set, a validation set, and a test set at a ratio of 8:1:1. After augmentation, 7677 images were used. A confusion matrix is a numerical table used to display the performance results of a classification model on test data with known target labels. It serves as a visual representation of how the model makes predictions on the test dataset. On the basis of the results obtained from each network validation, we plotted the normalized confusion matrix, with the true labels on the vertical axis and the predicted labels on the horizontal axis (Figures 5-8). Table 4 provides a summary of the accuracy, precision, recall, and *F*_1_-scores for the 4 CNNs.

**Table 4.** The model’s overall classification performance.

Modes and PI^a^ stages		Accuracy (%)	Precision (%)	Recall(%)	*F*_1_-score (%)
AlexNet					
	Stage I	92.76	98.25	91.43	94.71
	Stage II	86.36	97.60	92.76	95.12
	Stage III	73.91	95.53	86.36	90.71
	Stage IV	89.89	99.00	73.91	84.63
	Unstageable	79.38	98.29	89.89	93.90
	SDTI^b^	91.43	96.24	79.38	87.00
	Overall	87.74	97.48	85.62	94.71
VGGNet 16					
	Stage I	96.19	99.19	96.19	97.66
	Stage II	80.26	93.78	93.78	86.49
	Stage III	82.47	94.36	94.36	88.01
	Stage IV	76.40	98.50	98.50	75.24
	Unstageable	81.44	96.17	96.17	85.15
	SDTI	96.19	96.38	96.38	88.28
	Overall	82.42	96.40	95.90	97.66
ResNet 18					
	Stage I	94.08	99.41	97.14	98.26
	Stage II	90.91	98.06	94.08	96.02
	Stage III	86.96	97.03	90.91	93.84
	Stage IV	92.13	99.50	86.96	92.80
	Unstageable	88.66	98.69	92.13	95.29
	SDTI	97.14	97.91	88.66	93.95
	Overall	92.42	98.43	91.64	98.26
DenseNet121					
	Stage I	93.42	99.03	95.24	97.09
	Stage II	96.10	97.88	93.42	95.59
	Stage III	78.26	98.70	96.10	97.38
	Stage IV	94.38	99.17	78.26	87.48
	Unstageable	91.75	99.06	94.38	96.66
	SDTI	95.24	98.47	91.75	94.99
	Overall	93.71	98.72	91.53	97.09

a PI: pressure injury.

b SDTI: suspected deep tissue injury.

## Discussion

### Principal Findings

Among all the CNN models, DenseNet121 demonstrated the highest overall accuracy of 93.71%. The classification performances of AlexNet, VGGNet16, and ResNet18 exhibited overall accuracies of 87.74%, 82.42%, and 92.42%, respectively.

PIs are a global health issue, and effective treatment requires early and accurate classification and prevention. The staging of the PI is usually a subjective evaluation by medical professionals via standard systems, but it can differ due to variations in staff experience, training, and wound characteristics [22].

CNNs are feedforward neural networks with convolutional computations and deep structures. They capture local image features regardless of position through their core convolutional operation. This allows CNN-based models to potentially reduce assessment disparities among medical personnel. This approach promises precise classification of PIs, ensuring consistent patient care and valuable advice post discharge, which improves quality of life and health care resource efficiency [23].

However, many factors need to be considered when a reliable staging model is constructed (eg, the quality and quantity of images matter for model training). Several previous studies were based on retrospectively collected images in which other types of wounds were included, and the shooting equipment may not have been updated with lower image pixels, which affects the reliability of supervised learning in DL [24-26]. Some researchers have attempted to classify PIs via neural network models on the basis of the clinical staging system; however, most of these studies have relied on public datasets containing a limited number of images per grade, typically only a few dozen [25,26]. It is recommended that more than 150 training images should be used per grade to achieve reasonable classification accuracy when resources are limited [27].

Therefore, it is necessary to construct a large dataset of high quality with reliable labeling of each grade. In contrast, in this study, PI images were collected prospectively by professional cameras, and a gray card and executable shooting standard were developed to ensure that the quality of each image was clear and that the wound characteristics could be clearly displayed. Moreover, the staging results of each image were assessed by 2 wound therapists simultaneously. The wound therapists included were all qualified and had more than 15 years of working experience, so their assessment results were convincing, which contributed to the good comparability of the images and the reliable sample set. Additionally, we used a professional digital camera and a gray card, which were rarely used in previous studies, to ensure the quality of the images, particularly in accurately representing the original color of PIs [28].

Compared with other studies, this study still has advantages in terms of classification accuracy. Ay et al [24] used the European Pressure Ulcer Advisory Panel staging system and included PIs from stage 1 to stage 4. They trained 1091 images from a public dataset of PIs called the PIID (Public Injury Images Dataset) and 15 images from Google via DenseNet121, InceptionV3, MobileNetV2, ResNet152, ResNet50, and VGG16. The results indicated that the average accuracy of the 6 algorithms for each stage during pretraining ranged from 54.84% to 77.42% [24]. Kim et al [29] set SE-ResNext101 to train 2614 images from 493 participants. The accuracy of the model was 0.793 over the internal testing set and 0.717 over the external testing set [29]. In our study, DenseNet121 exhibited the highest accuracy (93.71%), precision (98.72%), recall (91.53%), and *F*_1_-score (97.09%), possibly because of the following reasons. (1) DenseNet121 improves the backpropagation of gradients due to the dense connection mode, making the network easier to train. Moreover, it can reduce the gradient disappearance problem caused by the transmission of input information and gradient information between many layers. (2) The number of parameters is reduced. (3) Low-dimensional features are preserved. In a standard convolutional network, the final output is only used to extract the highest-level features [30]. It is also important to consider the algorithm and image quality. In our study, 5 wound therapists, all of whom were qualified and had been engaged in wound management for at least 15 years, were recruited for grading. Therefore, the results of image staging were relatively accurate, and the classification of each label learned by CNNs was also convincing. Overall, DenseNet121 has become a popular choice in the field of computer vision because of its effective training of deep networks, high performance, generalizability, and insights into the learning process. These advantages make them powerful tools for image recognition and classification tasks. However, DenseNet121 processes a large number of layers, is relatively time-consuming, and consumes considerable computing power. DenseNet121 is more suitable for our present small sample. In regard to large-scale datasets, from the perspective of computing power and time factors, ResNet-18 or a higher level, such as ResNet34 or ResNet50, may be a better choice [31].

To improve the model’s adaptability to skin color variations, future research should increase the sample size and expand collection areas. Additionally, implementing the model in clinical settings requires effective communication due to the perception of opacity in DL methods. Accountability is crucial in the medical field, as errors can have legal implications.

### Limitations

The images obtained from data segmentation in this study contain some normal skin tissue. The next step is to make the wound more prominent or to make a judgment after segmenting the image.

Clinical wound management is influenced by blood and fluid seepage. Future studies should integrate DL with 3D imaging, thermal imaging, and fluid seepage assessment for a more comprehensive wound assessment.

The study also faced challenges due to limited prospective image data and reliance on a single device, potentially leading to biases and affecting model accuracy. This study did not compare the model’s performance with that of wound therapists or nurses in discriminating pressure ulcers; instead, it focused on internal validation and algorithm comparison.

### Conclusions

Staging models that use CNNs as their foundation exhibit robust classification capabilities. However, further research is needed to validate the reliability of these observed results. As such, we intend to gather an extensive array of additional imagery and undertake a comparative analysis between the staging outcomes generated by the model and those achieved by frontline clinical nurses. This comparative assessment will allow us to identify any potential discrepancies and disparities between the two, thereby affording us valuable insights for refining the model’s performance and suggesting effective strategies for enhancing the skills and capabilities of nurses.

## Abbreviations

CNN: convolutional neural network
DL: deep learning
NPIAP: National Pressure Injury Advisory Panel
PI: pressure injury
PIID: Public Injury Images Dataset
SDTI: suspected deep tissue injury
